# The genome sequence of the marbled white butterfly, *Melanargia galathea *(Linnaeus, 1758)

**DOI:** 10.12688/wellcomeopenres.17807.1

**Published:** 2022-04-04

**Authors:** Roger Vila, Konrad Lohse, Alex Hayward, Dominik Laetsch, Natural History Museum Genome Acquisition Lab, Darwin Tree of Life Barcoding collective, Wellcome Sanger Institute Tree of Life programme, Wellcome Sanger Institute Scientific Operations: DNA Pipelines collective, Tree of Life Core Informatics collective, Darwin Tree of Life Consortium

**Affiliations:** 1 Institut de Biologia Evolutiva (CSIC - Universitat Pompeu Fabra), Barcelona, Spain; 2 Institute of Evolutionary Biology, University of Edinburgh, Edinburgh, UK; 3 College of Life and Environmental Sciences, Department of Biosciences, University of Exeter, Penryn, UK

**Keywords:** Melanargia galathea, marbled white, genome sequence, chromosomal, Lepidoptera

## Abstract

We present a genome assembly from an individual female *Melanargia galathea *(the marbled white; Arthropoda; Insecta; Lepidoptera; Nymphalidae). The genome sequence is 606 megabases in span. The majority (99.97%) of the assembly is scaffolded into 25 chromosomal pseudomolecules, with the W and Z sex chromosomes assembled.

## Species taxonomy

Eukaryota; Metazoa; Ecdysozoa; Arthropoda; Hexapoda; Insecta; Pterygota; Neoptera; Endopterygota; Lepidoptera; Glossata; Ditrysia; Papilionoidea; Nymphalidae; Satyrinae; Satyrini; Melanargiina; *Melanargia*; *Melanargia galathea* (Linnaeus, 1758) (NCBI:txid111923).

## Background

The marbled white *Melanargia galathea* is a common butterfly of flower-rich meadows and other grassy habitats in central and southern Europe, Caucasus, Transcaucasia and northern and central parts of Asia Minor. The species is notably absent from most Mediterranean islands, from most of the Iberian peninsula (where it is replaced by the closely related *Melanargia lachesis*) and from northwestern Africa (where it is replaced by *Melanargia lucasi*) ( Habel *et al.*, 2017). *Melanargia galathea* is univoltine, with a flight period from late May to September depending on latitude. Early instar larvae overwinter and can feed on a wide range of grasses. Wing patterns vary throughout the range and several discrete varieties have been described as forms, in particular darker forms (f. *procida* and f. *magdalenae*) and specimens with the hind wing underside uniformly white, unmarked (f. *leucomelas*) ( Tolman & Lewington, 2008).

*Melanargia galathea* is currently listed as a species of Least Concern in the IUCN Red List of Europe ( van Swaay *et al.*, 2010). UK populations feed mainly on Red Fescue ( *Festuca rubra*) and have been classified as ssp. *serena*, based on subtle wing pattern differences ( Verity, 1913). While *M. galathe*a is restricted to England and Wales in the UK, it has expanded its range rapidly northwards in recent decades ( Fox *et al.*, 2015). Successful introductions to previously unoccupied sites in Northern England suggest that the species lags behind its current climatic niche at the range margin ( Willis *et al.*, 2009). The species has a karyotype of 24 chromosomes ( Bigger, 1960; Lorković, 1941).

## Genome sequence report

**Figure 1.**
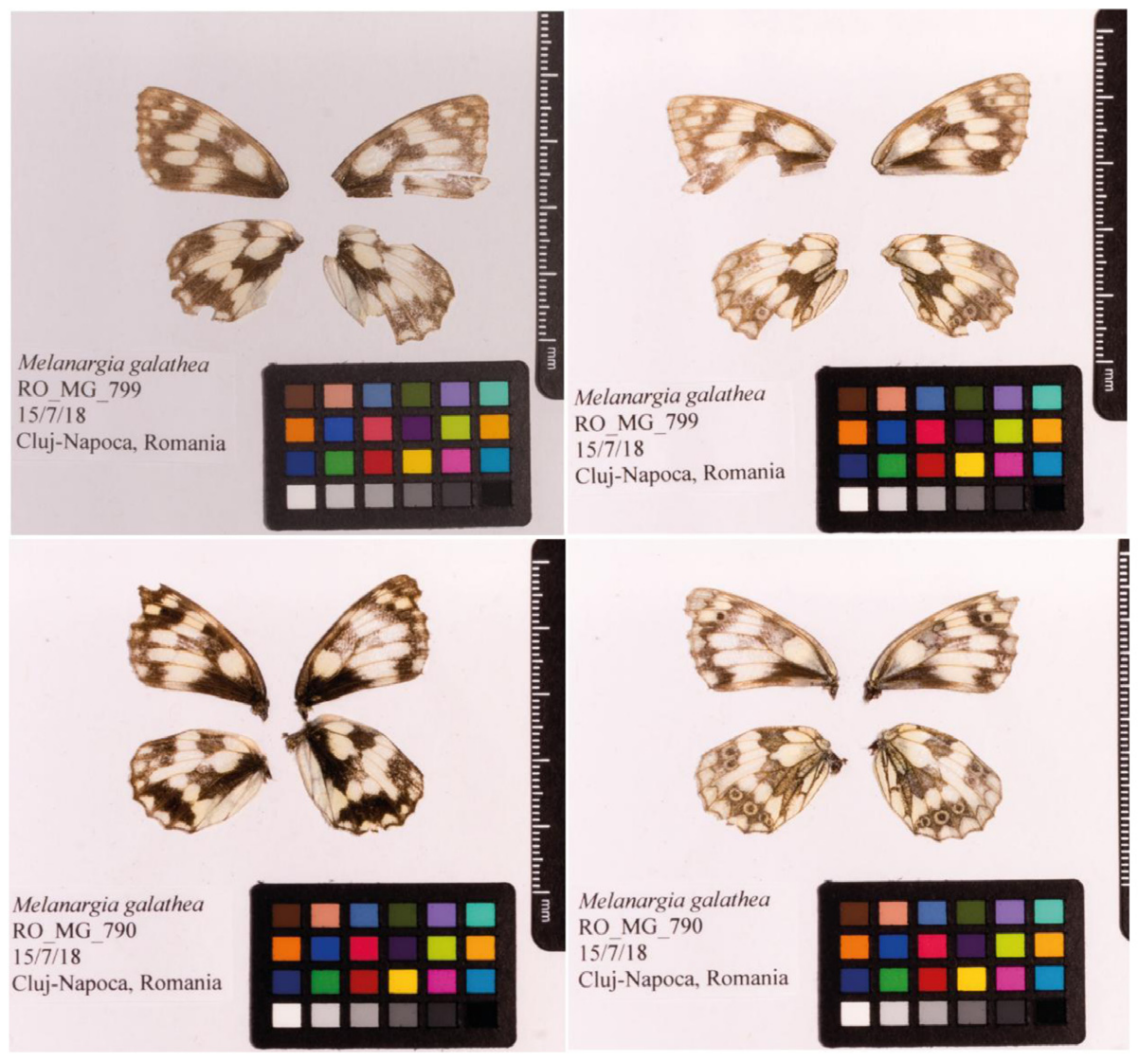
Fore and hind wings of the *Melanargia galathea* specimen from which the genome was sequenced. Dorsal (left) and ventral (right) surface view of wings from specimen RO_MG_799 (ilMelGala2) from Cluj-Napoca, Romania, used to generate Pacific Biosciences and 10X genomics data. Dorsal (left) and ventral (right) surface view of wings from specimen RO_MG_790 (ilMelGala1) from Cluj-Napoca, Romania, used to generate Hi-C data.

The genome was sequenced from a single female *M. galathea* ( Figure 1) collected near Cluj Napoca, Romania (latitude 46.834, longitude 23.629). A total of 29-fold coverage in Pacific Biosciences single-molecule circular consensus (HiFi) long reads and 55-fold coverage in 10X Genomics read clouds were generated. Primary assembly contigs were scaffolded with chromosome conformation Hi-C data. Manual assembly curation corrected 69 missing/misjoins and removed 7 haplotypic duplications, reducing the assembly length by 0.63% and the scaffold number by 45.36%, and increased the scaffold N50 by 23.78%.

**Figure 2.**
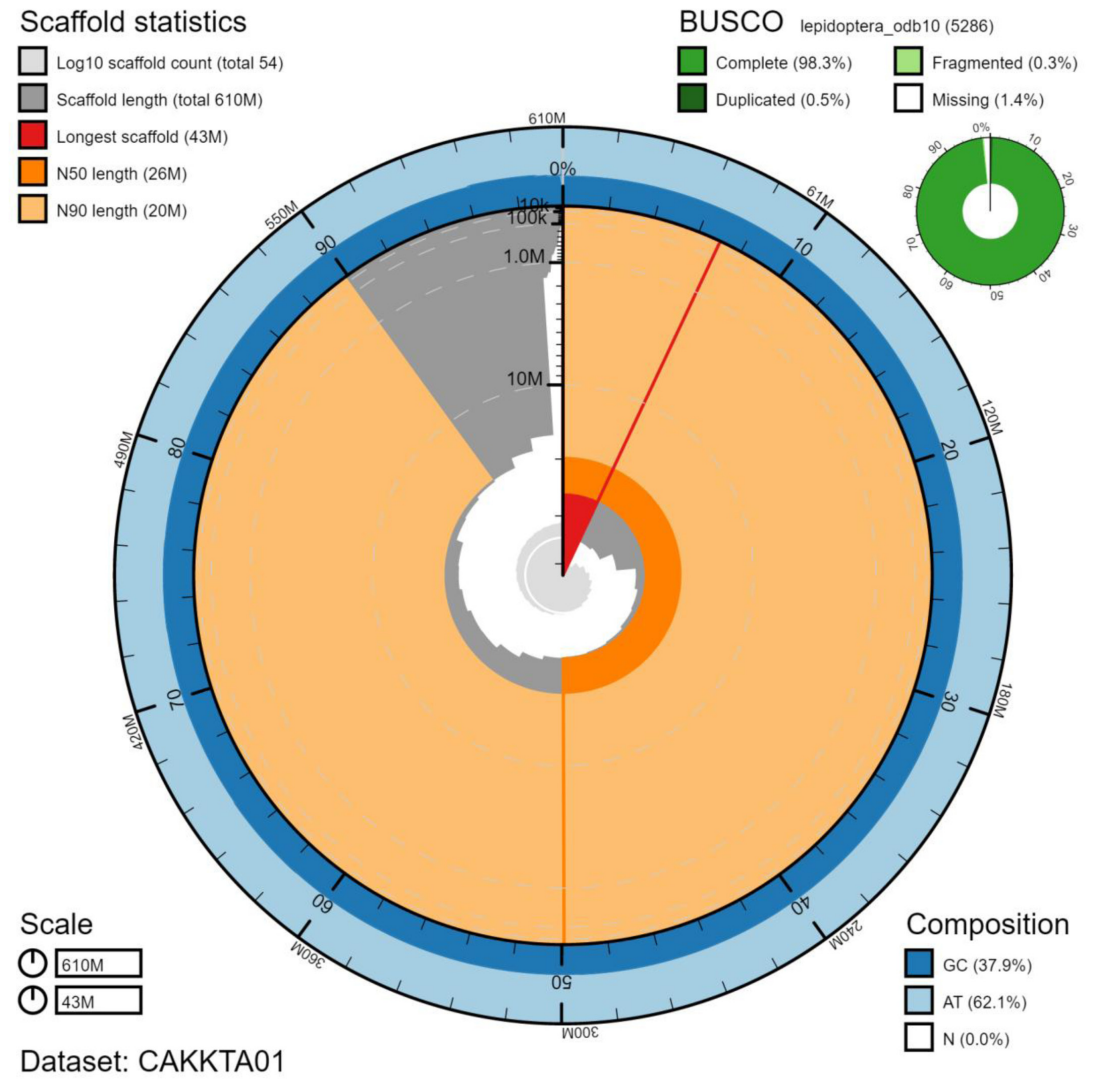
Genome assembly of *Melanargia galathea*, ilMelGala2.1: metrics. The BlobToolKit Snailplot shows N50 metrics and BUSCO gene completeness. The main plot is divided into 1,000 size-ordered bins around the circumference with each bin representing 0.1% of the 606,376,002 bp assembly. The distribution of chromosome lengths is shown in dark grey with the plot radius scaled to the longest chromosome present in the assembly (42,613,449 bp, shown in red). Orange and pale-orange arcs show the N50 and N90 chromosome lengths (25,507,680 and 19,649,718 bp), respectively. The pale grey spiral shows the cumulative chromosome count on a log scale with white scale lines showing successive orders of magnitude. The blue and pale-blue area around the outside of the plot shows the distribution of GC, AT and N percentages in the same bins as the inner plot. A summary of complete, fragmented, duplicated and missing BUSCO genes in the lepidoptera_odb10 set is shown in the top right. An interactive version of this figure is available at https://blobtoolkit.genomehubs.org/view/ilMelGala2.1/dataset/CAKKTA01/snail.

The final assembly has a total length of 606 Mb in 53 sequence scaffolds with a scaffold N50 of 25.5 Mb ( Table 1). The majority, 99.97%, of assembly sequence was assigned to 25 chromosomal-level scaffolds, representing 23 autosomes (numbered by sequence length), and the W and Z sex chromosome ( Figure 2– Figure 5; Table 2). The assembly has a BUSCO v5.1.2 ( Manni *et al.*, 2021) completeness of 98.3% (single 97.8%, duplicated 0.5%) using the lepidoptera_odb10 reference set (n=5286). While not fully phased, the assembly deposited is of one haplotype. Contigs corresponding to the second haplotype have also been deposited.

**Table 1.** Genome data for *Melanargia galathea*, ilMelGala2.1.

*Project accession data*	
Assembly identifier	ilMelGala2.1
Species	*Melanargia galathea*
Specimen	ilMelGala2 (genome assembly); ilMelGala1 (Hi-C, additional HiFi and 10X reads); ilMelGala4 (RNA-Seq)
NCBI taxonomy ID	NCBI:txid111923
BioProject	PRJEB46857
BioSample ID	SAMEA7523296
Isolate information	Female, whole organisms (ilMelGala1, ilMelGala2); unknown sex, whole organism (ilMelGala4)
*Raw data accessions*	
PacificBiosciences SEQUEL II	ERR6808026 (ilMelGala2), ERR7224283 (ilMelGala1)
10X Genomics Illumina	ERR6688677-ERR6688680 (ilMelGala2); ERR6688667-ERR6688670, ERR6688672- ERR6688675 (ilMelGala1)
Hi-C Illumina	ERR6688671
Illumina polyA RNA-Seq	ERR6688676
*Genome assembly*	
Assembly accession	GCA_920104075.1
*Accession of alternate* *haplotype*	GCA_920103875.1
Span (Mb)	606
Number of contigs	147
Contig N50 length (Mb)	9.2
Number of scaffolds	53
Scaffold N50 length (Mb)	25.5
Longest scaffold (Mb)	42.6
BUSCO * genome score	C:98.3%[S:97.8%,D:0.5%],F:0.3%,M:1.4%,n:5286

*BUSCO scores based on the lepidoptera_odb10 BUSCO set using v5.1.2. C= complete [S= single copy, D=duplicated], F=fragmented, M=missing, n=number of orthologues in comparison. A full set of BUSCO scores is available at https://blobtoolkit.genomehubs.org/view/ilMelGala2.1/dataset/CAKKTA01/busco.

**Figure 3.**
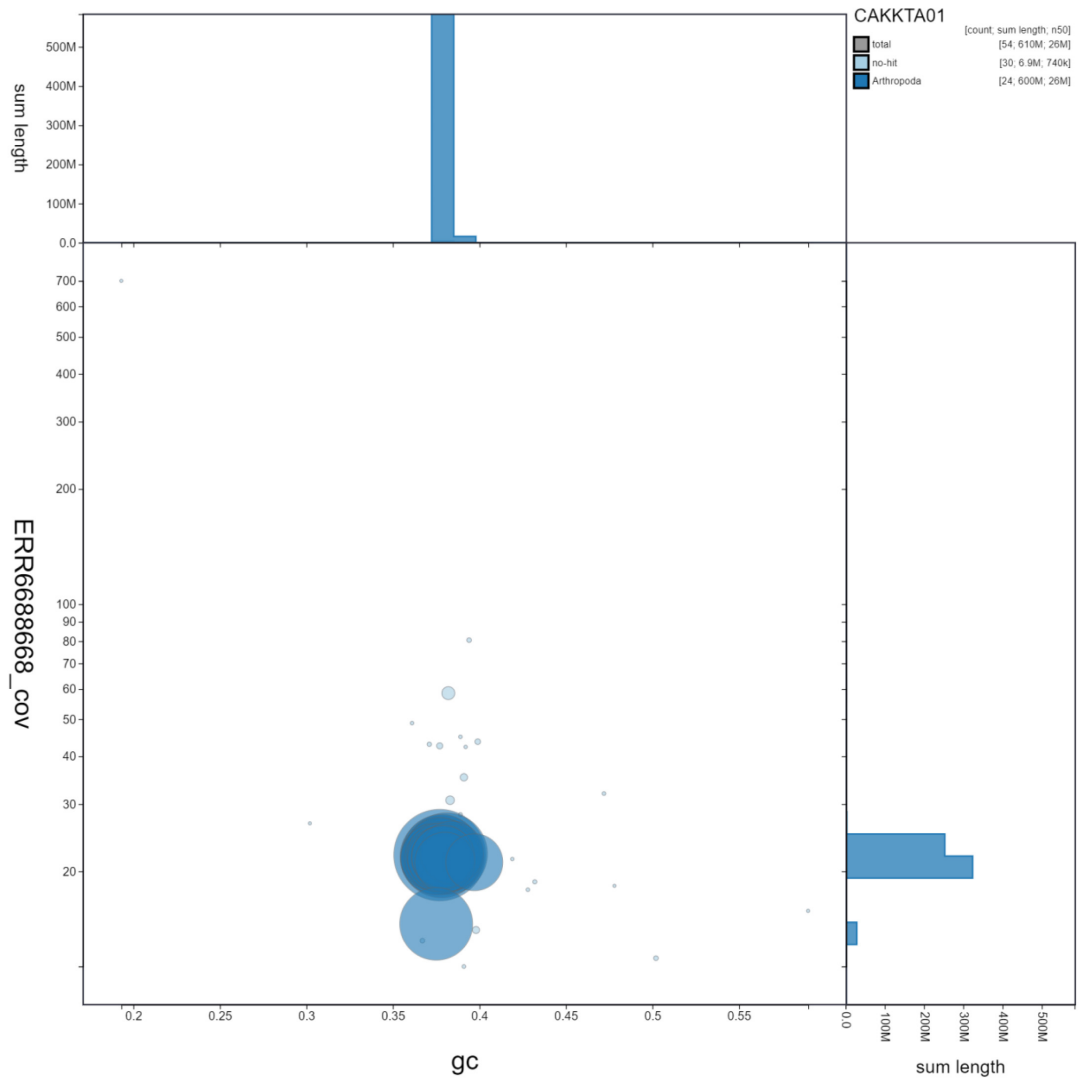
Genome assembly of *Melanargia galathea*, ilMelGala2.1: GC coverage. BlobToolKit GC-coverage plot. Scaffolds are coloured by phylum. Circles are sized in proportion to scaffold length. Histograms show the distribution of scaffold length sum along each axis. An interactive version of this figure is available at https://blobtoolkit.genomehubs.org/view/ilMelGala2.1/dataset/CAKKTA01/blob.

**Figure 4.**
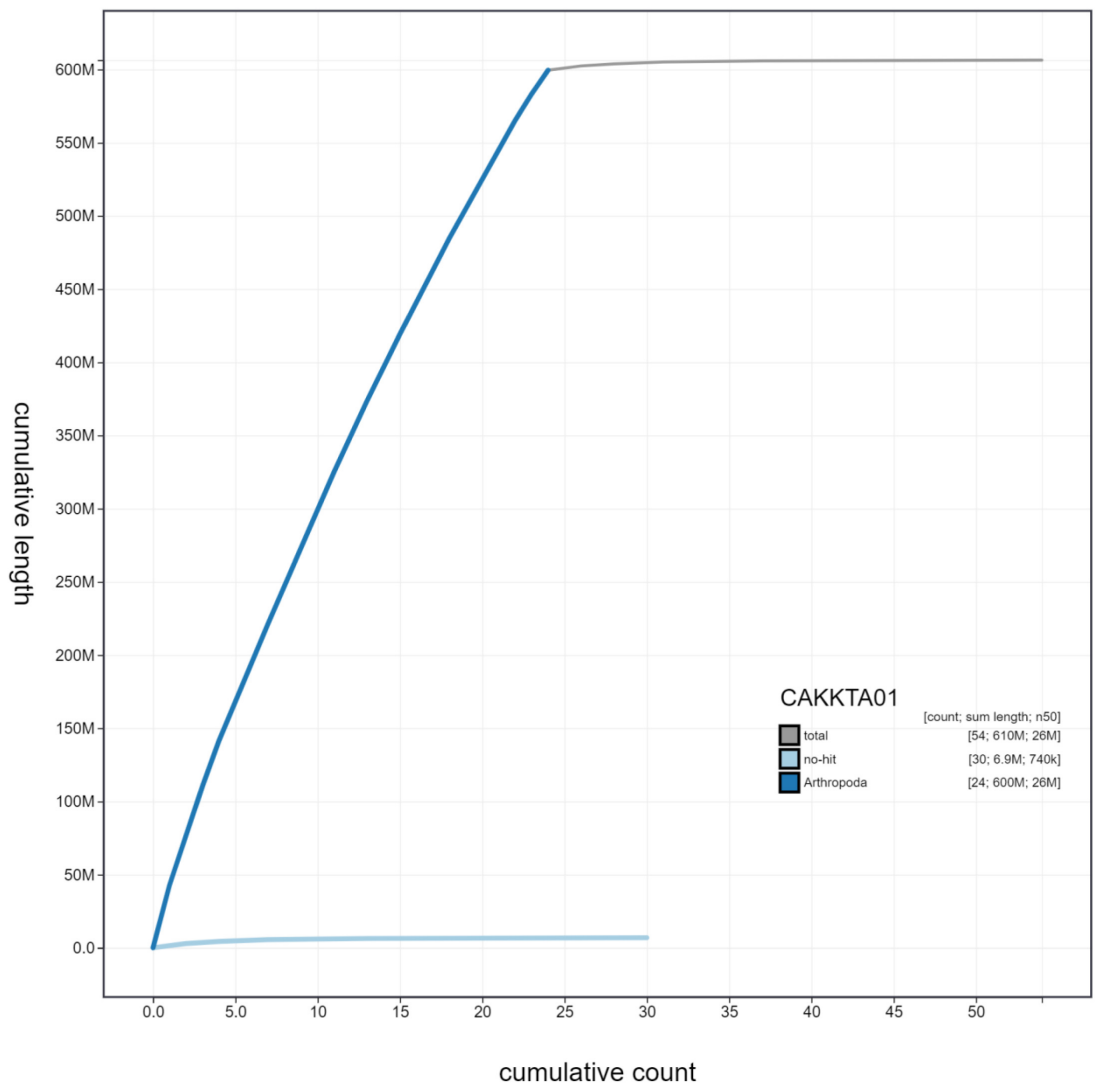
Genome assembly of *Melanargia galathea*, ilMelGala2.1: cumulative sequence. BlobToolKit cumulative sequence plot. The grey line shows cumulative length for all scaffolds. Coloured lines show cumulative lengths of scaffolds assigned to each phylum using the buscogenes taxrule. An interactive version of this figure is available at https://blobtoolkit.genomehubs.org/view/ilMelGala2.1/dataset/CAKKTA01/cumulative.

**Figure 5.**
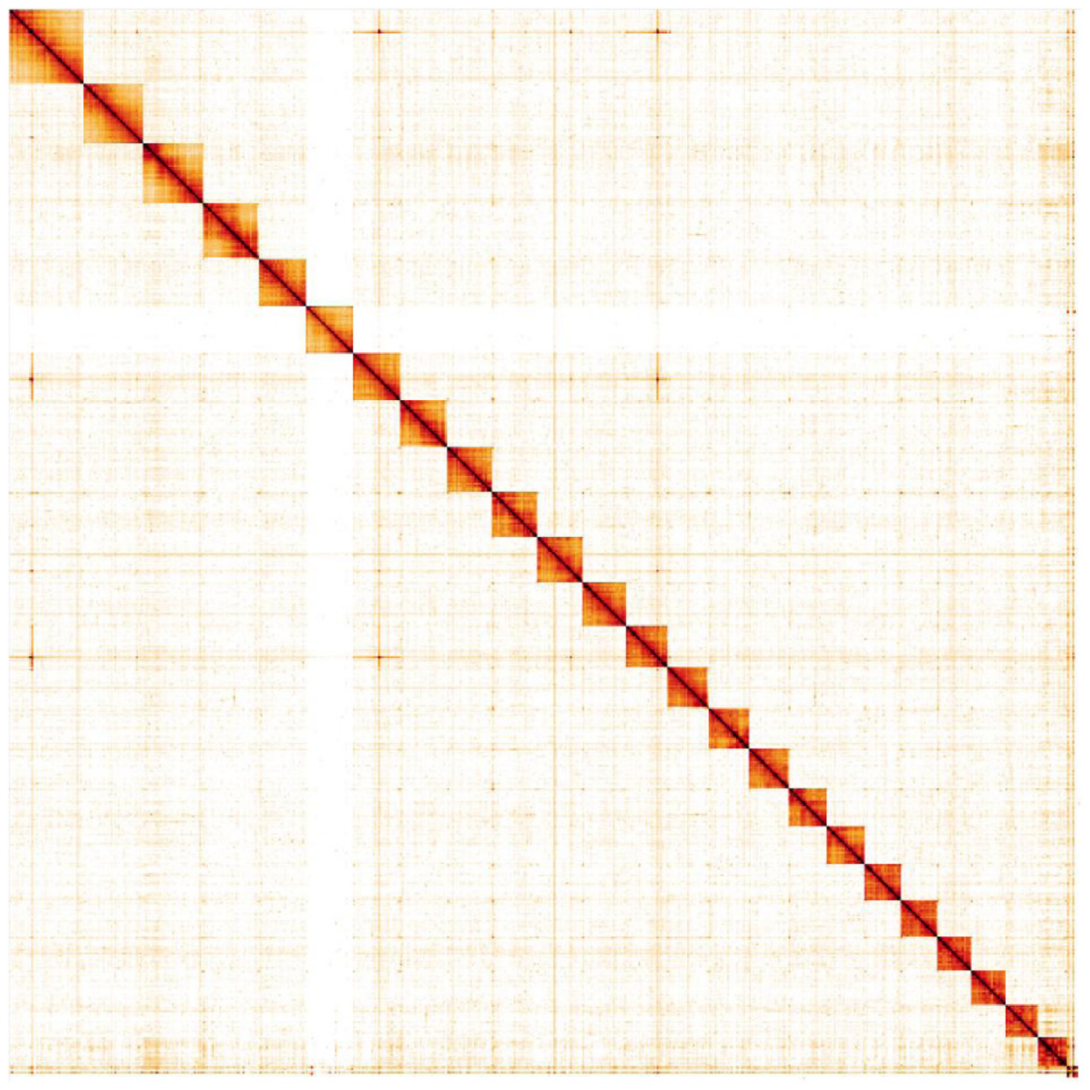
Genome assembly of *Melanargia galathea*, ilMelGala2.1: Hi-C contact map. Hi-C contact map of the ilMelGala2.1 assembly, visualised in HiGlass. Chromosomes are shown in size order from left to right and top to bottom. The interactive Hi-C map can be viewed here.

**Table 2.** Chromosomal pseudomolecules in the genome assembly of *Melanargia galathea*, ilMelGala2.1.

INSDC accession	Chromosome	Size (Mb)	GC%
OV049855.1	1	42.61	37.7
OV049856.1	2	34.19	37.8
OV049857.1	3	33.66	38.0
OV049858.1	4	30.96	37.8
OV049859.1	5	27.26	37.6
OV049861.1	6	26.49	37.7
OV049862.1	7	26.08	37.7
OV049863.1	8	25.86	37.9
OV049864.1	9	25.72	37.9
OV049865.1	10	25.51	37.5
OV049866.1	11	24.81	37.6
OV049867.1	12	23.74	37.7
OV049868.1	13	22.82	38.3
OV049869.1	14	22.76	37.4
OV049870.1	15	22.18	37.6
OV049871.1	16	21.78	38.2
OV049872.1	17	21.66	37.7
OV049873.1	18	20.52	37.7
OV049874.1	19	20.32	37.9
OV049875.1	20	19.93	38.4
OV049876.1	21	19.65	37.9
OV049877.1	22	18.08	38.0
OV049878.1	23	16.22	39.7
OV049879.1	W	1.54	38.5
OV049860.1	Z	26.70	37.5
OV049880.1	MT	0.02	19.6
-	Unplaced	5.31	38.7

## Methods

### Sample acquisition and nucleic acid extraction

Two *M. galathea* specimens (ilMelGala2, genome assembly; ilMelGala1, Hi-C, additional HiFi and 10X reads not used in genome assembly; ilMelGala4) were collected near Cluj Napoca, Romania (latitude 46.834, longitude 23.629) using a net by Konrad Lohse, Alex Hayward, Dominik Laetsch and Roger Vila, who also identified the samples. The samples were snap-frozen at -80°C. A further specimen (ilMelGala4, RNA-Seq) was collected from Wigmore Park, Percival Way, Wigmore, Luton, UK by Olga Sivell, Natural History Museum, using a net. The specimen was identified by the same individual and snap-frozen on dry ice.

DNA was extracted from the whole organism of ilMelGala1 and ilMelGala2 at the Wellcome Sanger Institute Tree of Life laboratory, Wellcome Sanger Institute. The samples were weighed and dissected on dry ice, with ilMelGala1 tissue set aside for Hi-C sequencing. Whole organism tissue of ilMelGala2 was cryogenically disrupted to a fine powder using a Covaris cryoPREP Automated Dry Pulveriser, receiving multiple impacts. Whole organism tissue of ilMelGala1 was disrupted using a Nippi Powermasher fitted with a BioMasher pestle. Fragment size analysis of 0.01-0.5 ng of DNA was then performed using an Agilent FemtoPulse. High molecular weight (HMW) DNA was extracted using the Qiagen MagAttract HMW DNA extraction kit. Low molecular weight DNA was removed from a 200-ng aliquot of extracted DNA using 0.8X AMpure XP purification kit prior to 10X Chromium sequencing; a minimum of 50 ng DNA was submitted for 10X sequencing. HMW DNA was sheared into an average fragment size between 12-20 kb in a Megaruptor 3 system with speed setting 30. Sheared DNA was purified by solid-phase reversible immobilisation using AMPure PB beads with a 1.8X ratio of beads to sample to remove the shorter fragments and concentrate the DNA sample. The concentration of the sheared and purified DNA was assessed using a Nanodrop spectrophotometer and Qubit Fluorometer and Qubit dsDNA High Sensitivity Assay kit. Fragment size distribution was evaluated by running the sample on the FemtoPulse system.

RNA (from the whole organism of ilMelGala4) was extracted in the Tree of Life Laboratory at the WSI using TRIzol, according to the manufacturer’s instructions. RNA was then eluted in 50 μl RNAse-free water and its concentration RNA assessed using a Nanodrop spectrophotometer and Qubit Fluorometer using the Qubit RNA Broad-Range (BR) Assay kit. Analysis of the integrity of the RNA was done using Agilent RNA 6000 Pico Kit and Eukaryotic Total RNA assay.

### Sequencing

Pacific Biosciences HiFi circular consensus and 10X Genomics read cloud DNA sequencing libraries were constructed according to the manufacturers’ instructions. Poly(A) RNA-Seq libraries were constructed using the NEB Ultra II RNA Library Prep kit. DNA and RNA sequencing was performed by the Scientific Operations core at the WSI on Pacific Biosciences SEQUEL II (HiFi), Illumina NovaSeq 6000 (ilMelGala2, 10X), HiSeq X (ilMelGala1, 10X) and Illumina HiSeq 4000 (RNA-Seq) instruments. Hi-C data were also generated from remaining whole organism tissue of ilMelGala1 using the Arima v1 Hi-C kit and sequenced on HiSeq X.

### Genome assembly

Assembly was carried out with Hifiasm ( Cheng *et al.*, 2021); haplotypic duplication was identified and removed with purge_dups ( Guan *et al.*, 2020). One round of polishing was performed by aligning 10X Genomics read data to the assembly with longranger align, calling variants with freebayes ( Garrison & Marth, 2012). The assembly was then scaffolded with Hi-C data ( Rao *et al.*, 2014) using SALSA2 ( Ghurye *et al.*, 2019). The assembly was checked for contamination and corrected using the gEVAL system ( Chow *et al.*, 2016) as described previously ( Howe *et al.*, 2021). Manual curation ( Howe *et al.*, 2021) was performed using gEVAL, HiGlass ( Kerpedjiev *et al.*, 2018) and Pretext. The mitochondrial genome was assembled using MitoHiFi ( Uliano-Silva *et al.*, 2021), which performed annotation using MitoFinder ( Allio *et al.*, 2020). The genome was analysed and BUSCO scores generated within the BlobToolKit environment ( Challis *et al.*, 2020). Table 3 contains a list of all software tool versions used, where appropriate.

**Table 3.** Software tools used.

Software tool	Version	Source
Hifiasm	0.15.3	Cheng *et al.*, 2021
purge_dups	1.2.3	Guan *et al.*, 2020
SALSA2	2.2	Ghurye *et al.*, 2019
longranger align	2.2.2	https://support.10xgenomics.com/genome-exome/software/pipelines/latest/advanced/other-pipelines
freebayes	1.3.1-17- gaa2ace8	Garrison & Marth, 2012
MitoHiFi	2	Uliano-Silva *et al.*, 2021
gEVAL	N/A	Chow *et al.*, 2016
HiGlass	1.11.6	Kerpedjiev *et al.*, 2018
PretextView	0.2.x	https://github.com/wtsi-hpag/PretextView
BlobToolKit	2.6.4	Challis *et al.*, 2020

## Data availability

European Nucleotide Archive: Melanargia galathea (marbled white). Accession number PRJEB46857; https://identifiers.org/ena.embl/PRJEB46857.

The genome sequence is released openly for reuse. The *M. galathea* genome sequencing initiative is part of the Darwin Tree of Life (DToL) project. The genome will be annotated using the RNA-Seq data and presented through the Ensembl pipeline at the European Bioinformatics Institute. All raw sequence data and the assembly have been deposited in INSDC databases. Raw data and assembly accession identifiers are reported in Table 1.

## Author information

Members of the Darwin Tree of Life Barcoding collective are listed here: https://doi.org/10.5281/zenodo.5744972.

Members of the Wellcome Sanger Institute Tree of Life programme are listed here: https://doi.org/10.5281/zenodo.6125027.

Members of Wellcome Sanger Institute Scientific Operations: DNA Pipelines collective are listed here: https://doi.org/10.5281/zenodo.5746904.

Members of the Tree of Life Core Informatics collective are listed here: https://doi.org/10.5281/zenodo.6125046.

Members of the Darwin Tree of Life Consortium are listed here: https://doi.org/10.5281/zenodo.5638618.
