# Supplementary material for: The genome sequence of the marbled white butterfly, Melanargia galathea (Linnaeus, 1758)
Source: Wellcome Open Res. 2022 Apr 4;7:123. [Version 1] doi: 10.12688/wellcomeopenres.17807.1 (PMC9975406; doi:10.12688/wellcomeopenres.17807.1)
Supplement: Peer review history

# Peer review: Reviewer response for version 1

**DOI:** 10.21956/wellcomeopenres.19714.r53743

**Published:** 2022-12-20

**Authors:** Linda Neaves

**Affiliations:** 1 Australian National University, Canberra, Australia

The article presents the genome assembly for the marbled white butterfly, *Melanargia galathea *(Linnaeus, 1758). The authors generated long-read Pacific Biosciences single-molecule circular consensus and 10X genomics data from a single female, while a second female from the same location is used to generate Hi-C data. A third individual, from a different location was used for RNA extraction. The authors assembled the genome sequence spanning 606 megabases, with the majority (99.97%) of the assembly was scaffolded into 25 chromosomal pseudomolecules, including W and Z sex chromosomes.

 The article clearly articulates the rationale for creating the dataset, and the methods are clearly presented, and appear appropriate. I think it could be a little clearer in the main text of the report section that separate individuals were used for PacBio and 10X genomics, and the Hi-C data. It is clear in Figure 1 and later in the methods sections that there were two individuals used.

 The authors mention a third individual was used to generate RNA-seq data. In the data availability section, it was indicated this would be used to annotate genome, but it isn’t clear in the methods or the main report text if or how the RNA data is used.

Are sufficient details of methods and materials provided to allow replication by others?

Yes

Is the rationale for creating the dataset(s) clearly described?

Yes

Are the datasets clearly presented in a useable and accessible format?

Yes

Are the protocols appropriate and is the work technically sound?

Yes

Reviewer Expertise:

Conservation genomics, NGS, evolution, molecular biology

I confirm that I have read this submission and believe that I have an appropriate level of expertise to confirm that it is of an acceptable scientific standard.

# Peer review: Reviewer response for version 1

**DOI:** 10.21956/wellcomeopenres.19714.r49684

**Published:** 2022-05-12

**Authors:** Elsa Call

**Affiliations:** 1 Department of Biology, Lund University, Lund, Sweden

This article presents the genome of the marbled white butterfly, *Melanargia galathea *(Linnaeus, 1758). The authors used the whole body of one female specimen to generate Pacific Biosciences and 10X genomics data, and another female specimen to generate Hi-C data. In addition, the authors mentioned a third specimen (ilMelGala4) for RNA extraction. The authors sequenced and assembled the genome of 606 Mb. The majority (99.97%) of the assembly is scaffolded into 25 chromosomal-level scaffolds, including the assembled W and Z sex chromosomes.

 Two small remarks:

 Figure 1 presents 4 pictures including 4 wings (fore and hind wings). It is a bit confusing to show 2 specimens in the picture, while the first mention of figure 1 is after “a single female *M. galathea*” in the Genome sequence report section. While the legend of the picture makes it clear that 2 specimens were used (one to generate both Pacific Biosciences and 10X genomics data; the other to generate Hi-C data), the text is not that clear.

 It was a bit surprising that the specimen used for RNA extraction (ilMelGala4) was not mentioned, except during the method. It is not clear from the main text what was the use of this specimen and the RNA extraction generated.

Are sufficient details of methods and materials provided to allow replication by others?

Yes

Is the rationale for creating the dataset(s) clearly described?

Yes

Are the datasets clearly presented in a useable and accessible format?

Yes

Are the protocols appropriate and is the work technically sound?

Yes

Reviewer Expertise:

Genomics, Lepidotera, Molecular Biology, NGS, Evolution

I confirm that I have read this submission and believe that I have an appropriate level of expertise to confirm that it is of an acceptable scientific standard.
